# The Biosynthesis of Non-Endogenous Apocarotenoids in Transgenic *Nicotiana glauca*

**DOI:** 10.3390/metabo12070575

**Published:** 2022-06-22

**Authors:** Xin Huang, Lucía Morote, Changfu Zhu, Oussama Ahrazem, Teresa Capell, Paul Christou, Lourdes Gómez-Gómez

**Affiliations:** 1 Department of Plant Production and Forestry Science, University of Lleida-Agrotecnio CERCA Center, Av. Alcalde Rovira Roure, 191, 25198 Lleida, Spain; xin.huang@udl.cat (X.H.); teresa.capell@udl.cat (T.C.); paul.christou@udl.cat (P.C.); 2 Department of Science and Agroforestal Technology and Genetics, Botanical Institut, University of Castilla-La Mancha, Campus Universitario s/n, 02071 Albacete, Spain; lucia.morote@uclm.es (L.M.); oussama.ahrazem@uclm.es (O.A.); 3 School of Life Sciences, Changchun Normal University, Changchun 130032, China; 4 ICREA, Catalan Institute for Research and Advanced Studies, Passeig Lluís Companys 23, 08010 Barcelona, Spain

**Keywords:** apocarotenoids, CsCCD2L, BdCCD4.1, crocins, *Nicotiana glauca*, metabolic engineering

## Abstract

Crocins are high-value compounds with industrial and food applications. Saffron is currently the main source of these soluble pigments, but its high market price hinders its use by sectors, such as pharmaceutics. Enzymes involved in the production of these compounds have been identified in saffron, Buddleja, and gardenia. In this study, the enzyme from Buddleja, BdCCD4.1, was constitutively expressed in *Nicotiana glauca*, a tobacco species with carotenoid-pigmented petals. The transgenic lines produced significant levels of crocins in their leaves and petals. However, the accumulation of crocins was, in general, higher in the leaves than in the petals, reaching almost 302 µg/g DW. The production of crocins was associated with decreased levels of endogenous carotenoids, mainly β-carotene. The stability of crocins in leaf and petal tissues was evaluated after three years of storage, showing an average reduction of 58.06 ± 2.20% in the petals, and 78.37 ± 5.08% in the leaves. This study illustrates the use of BdCCD4.1 as an effective tool for crocin production in *N. glauca* and how the tissue has an important impact on the stability of produced high-value metabolites during storage.

## 1. Introduction

Carotenoids are isoprenoid pigments with multiple conjugated double bonds synthesized by plants, yeast, algae, bacteria, and some animals [1]. The basic structure of carotenoids is a C40 hydrocarbon skeleton of conjugated double bonds (the chromophore) and linear or cyclic end groups. Carotenoids are divided into carotenes, which contain only carbon and hydrogen atoms, and xanthophylls, which additionally contain oxygen atoms as different functional groups (keto, hydroxyl, epoxy, etc.) [2]. Plant carotenoids accumulate in plastids, where they participate as accessory pigments in photosynthesis and in the xanthophyll cycle, preventing photo-oxidative damage. In petals and fruits, carotenoids accumulate in chromoplasts, providing color and acting as precursors of aroma volatiles, thus facilitating pollination and seed dispersal [3]. The biosynthetic pathway of carotenoids in plants has been extensively reviewed [1,4]. Geranylgeranyl diphosphate (GGDP) is produced via the plastidial methylerythritol 4-phosphate (MEP) pathway [5]. GGDP is the substrate for the biosynthesis of phytoene, a non-colored carotenoid that, after a series of desaturation and isomerization reactions, is converted to all-trans-lycopene. Lycopene is the substrate for lycopene cyclase enzymes to form either β- or α-carotene. A sequence of hydroxylation and epoxidation reactions leads to the production of the xanthophylls, including lutein, zeaxanthin, antheraxanthin, and violaxanthin. In non-photosynthetic tissues, such as petals and fruits, xanthophylls are present as unconjugated molecules or as mono- or diesters [6], with implications for xanthophyll storage, retention, and cleavage.

The carotenoid backbone can be truncated by the enzymatic or non-enzymatic removal of fragments, generating apocarotenoids [7]. Based on the number of carbons and double bonds in the apocarotenoid skeleton, these compounds exhibit distinctive yellow, orange, and red colors, while these properties also determine their degree of volatility [8]. The biosynthesis of apocarotenoids in all living organisms is catalyzed by carotenoid-cleavage dioxygenase enzymes (CCDs), which differ in terms of their substrate specificity and cleavage position [8]. In plants, there are two major families of CCDs: 9-cis-epoxycarotenoid dioxygenase (NCED), involved in the biosynthesis of abscisic acid (ABA) [9]; and CCD, acting on carotenoid or apocarotenoid substrates and exhibiting diverse substrate specificity, regioselectivity, and physiological functions [8]. The apocarotenoids produced by the activity of CCD enzymes have a broad range of biological functions, such as phytohormones (e.g., abscisic acid and strigolactones) [10,11], signals in herbivore–plant communication [12], in microorganism–plant communication [11], and chromophores (e.g., bixin and crocins) [13].

**Figure 1:**
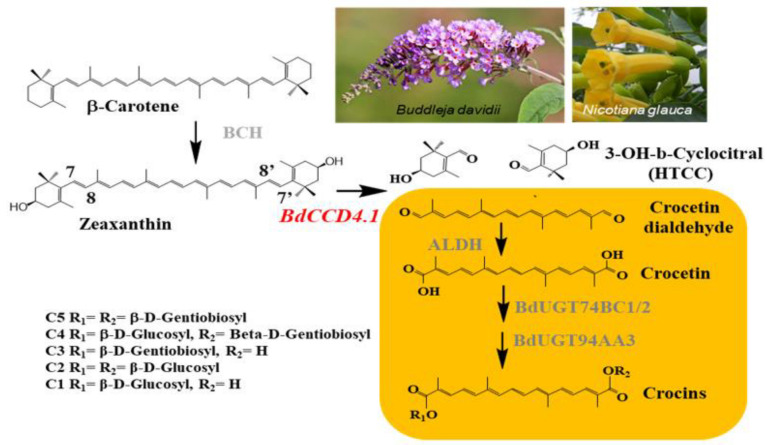
Biosynthetic pathway of crocins from zeaxanthin in *Buddleja davidii*. Pictures of *B. davidii* and *N. glauca* petals are shown in the upper right. The recombinant enzyme introduced in *N. glauca* under the control of the CaMV35S promoter is shown in red. The yellow background shows the stages of transformation of crocetindial into the different crocins present in the petals of *B. davidii*. The arrows show each of the conversion steps catalyzed by the corresponding enzyme in gray. The different substitutions for the crocins present in the petals of *B. davidii* are shown in the lower left.

Crocins are high-value apocarotenoids used in the food and pharmaceutical industries, with the main natural sources of these compounds being saffron [14], followed by gardenia [15]. However, in Europe, Canada, and the United States, saffron is the only authorized source for the culinary industry, although adulteration with gardenia extracts is often found [16]. Irrespectively, the uses of crocins are expanding beyond the food sector, e.g., in the medical and pharmaceutical industries, as dietary supplements, and in cosmetics [17]. New natural sources are required to satisfy this substantial increase in the demand for crocins [18]. Alternatively, the development of more cost-effective production strategies in heterologous systems by the expression of the gene encoding the key enzyme for crocin biosynthesis in saffron, CCD2, can also be considered in biotechnological applications. This approach has been exploited with varying degrees of success in bacteria [19], yeast [20], and in plants by using different species and transgenic approaches [21,22,23,24]. Among the natural sources of these compounds, *Buddleja davidii* accumulates crocins in its petals, but at much lower levels (ca. 10-fold lower) compared with the stigma of saffron [25]. Although these levels are much higher than those obtained using other heterologous systems, *B. davidii* is not readily amenable to agricultural production. Even though Buddleja may not be a suitable production host, its native biosynthetic pathway for crocin biosynthesis has been elucidated [18,25] (Figure 1), and the key CCD enzyme, BdCCD4.1, has been isolated [18], thus facilitating metabolic engineering in heterologous hosts [23]. Zeaxanthin is the precursor used by this enzyme in *B. davidii* (Figure 1). However, the ability of BdCCD4.1 to act on other plant-based carotenoids has not yet been explored. In this work, we expressed *BdCCD4.1* in *Nicotiana glauca* Graham under the control of a constitutive promoter and we studied the stability of the apocarotenoids produced in the leaves and petals. *N. glauca* had been used previously as a platform to produce ketocarotenoids [26], taking advantage of its highly pigmented petals that mainly accumulate lutein. In addition, *N. glauca* grows in arid environments [27], is usually not suitable for food crops, and has immense potential as a biofuel stock due to its exceptionally high content of hydrocarbons in the leaves [28,29]. Previously, we had used *N. glauca* to produce crocins constitutively expressing *CsCCD2L*, the gene encoding for the enzyme responsible for crocin biosynthesis in saffron [22,30]. Here, we compared the performance of BdCCD4.1 and CsCCD2L for the production of crocins in *N. glauca* as a novel host for value-added products, such as crocins, and studied the stability of these metabolites during storage.

## 2. Results

### 2.1. Generation of N. glauca Plants Expressing BdCCD4.1

*Agrobacterium*-mediated transformation was used to introduce *BdCCD4.1* into *N. glauca*. The transformation vector contained the *BdCCD4.1* coding sequence (KX374547) under the control of the CaMV35S promoter and the *Hph* gene for selection with the antibiotic hygromycin. Following antibiotic selection, plants confirmed to contain the transgene by PCR were regenerated. Five independent transformed lines were selected and grown in the greenhouse. Two non-transgenic (PCR-negative) plants were used as controls.

### 2.2. Levels of Crocins in Leaves and Petals of Transgenic N. glauca

**Figure 2:**
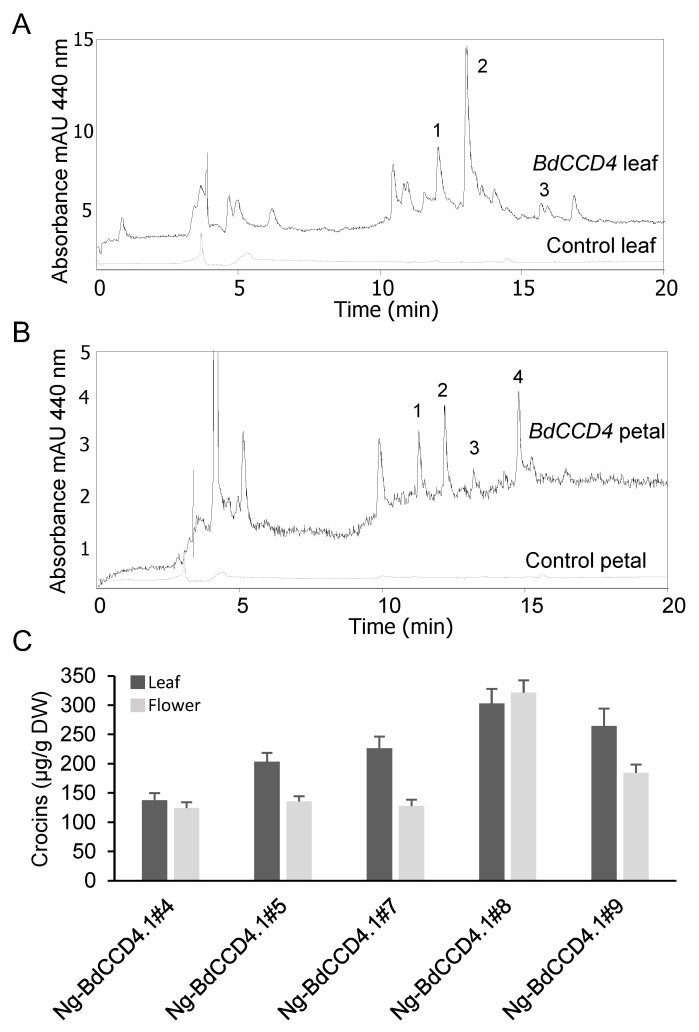
Accumulation of crocins in Wt and transgenic T1 lines of *Nicotiana glauca* constitutively expressing *BdCCD4.1*. (**A**) HPLC-DAD analysis of polar extracts of Wt and *N. glauca* leaves at 440 nm. (**B**) HPLC-DAD analysis of polar extracts from transgenic and Wt *N. glauca* petals at 440 nm. Peaks for the abundant crocins in the transgenic lines are denoted by numbers in leaves (1 = trans-crocin 3; 2 = trans-crocin 2; 3 = cis-crocin 2) and petals (1 = trans-crocin 4; 2 = trans-crocin 3; 3 = trans-crocin 2; 4 = cis-crocin 2); these crocins were completely absent from Wt plants. mAU, milli-absorbance units. (**C**) Apocarotenoid accumulation in the leaves and petals of transgenic lines. Analyses were conducted in triplicate. Error bars represent the SD. DW, dry weight.

**Figure 3:**
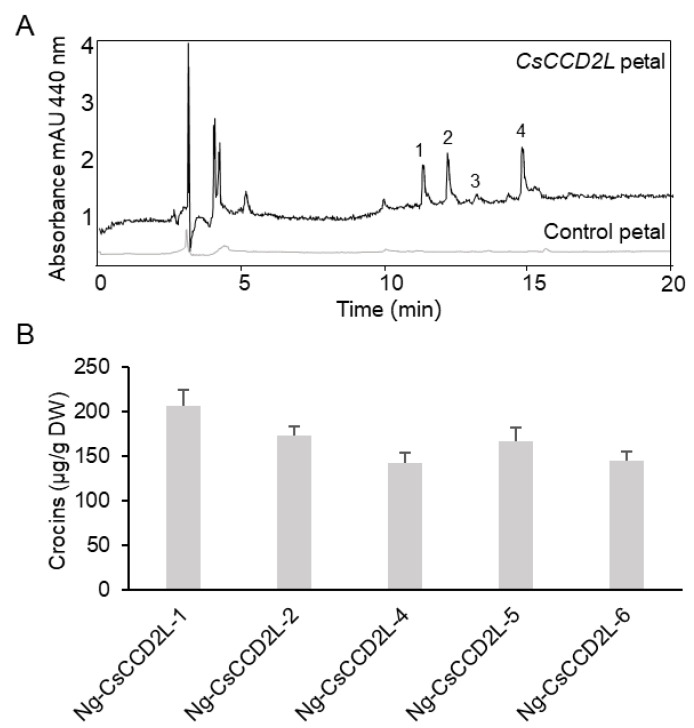
Accumulation of apocarotenoids in Wt and transgenic T1 lines of *Nicotiana glauca* expressing *CsCCD2L*. (**A**) HPLC-DAD analysis of polar extracts from Wt *N. glauca* petals at 440 nm. The peaks for the abundant crocins in the transgenic lines are as follows: 1 = trans-crocin 4; 2 = trans-crocin 3; 3 = trans-crocin 2; 4 = cis-crocin 2; crocins were completely absent from Wt plants. mAU, milli-absorbance units. (**B**) Apocarotenoid accumulation in the petals of transgenic lines. Analyses were conducted in triplicate. Error bars represent the SD. DW, dry weight.

In order to quantify apocarotenoids in the plants, a previously described HPLC system [22,23]) was used for the analyses of the polar extracts of leaves and petals. Typical chromatograms of the apocarotenoids formed in the leaves and petals of transgenic *N. glauca* plants expressing *BdCCD4.1* are shown in Figure 2. The profiles of crocins in the leaves and petals clearly differed between these tissues (Figure 2A,B), suggesting the presence of different tissue-specific endogenous glycosyltransferases. The predominant crocin moieties contained three and two glucose molecules. The amounts of crocins in petals and leaves in the transgenic lines are shown in Figure 2C. In three transgenic lines, the levels of crocins were higher in the leaves than in the petals (lines #5, #7, and #9). In the two transgenic lines, the crocin levels were similar in the leaves and petals (lines #1 and #8). The levels of crocins in the five lines we analyzed ranged from 137 to 302 µg/g. Previously, we analyzed the levels of crocins in *N. glauca* leaves from plants transformed with the saffron enzyme for crocin biosynthesis, *CsCCD2L*. The levels of crocins in the leaves of these plants were within the same order of magnitude, but higher than those obtained with *BdCCD4.1* (1.5 fold difference on average) [22]. We also analyzed the crocin content of the petals of lines we had generated previously expressing *CsCCD2L* [22]. Interestingly, the levels of crocins in the petals from transgenic *CsCCD2L* were lower, with a range of 56–86 µg/g (Figure 3), than in the leaves, with a range of 280–394 µg/g [22], suggesting a lower availability of substrates for CsCCD2L in the petals. In fact, zeaxanthin was found at higher levels in the leaves than in the petals in *N. glauca*, where lutein predominates [31].

### 2.3. Levels of Endogenous Carotenoids in Leaves and Petals of Transgenic N. glauca Expressing BdCCD4.1

**Figure 4:**
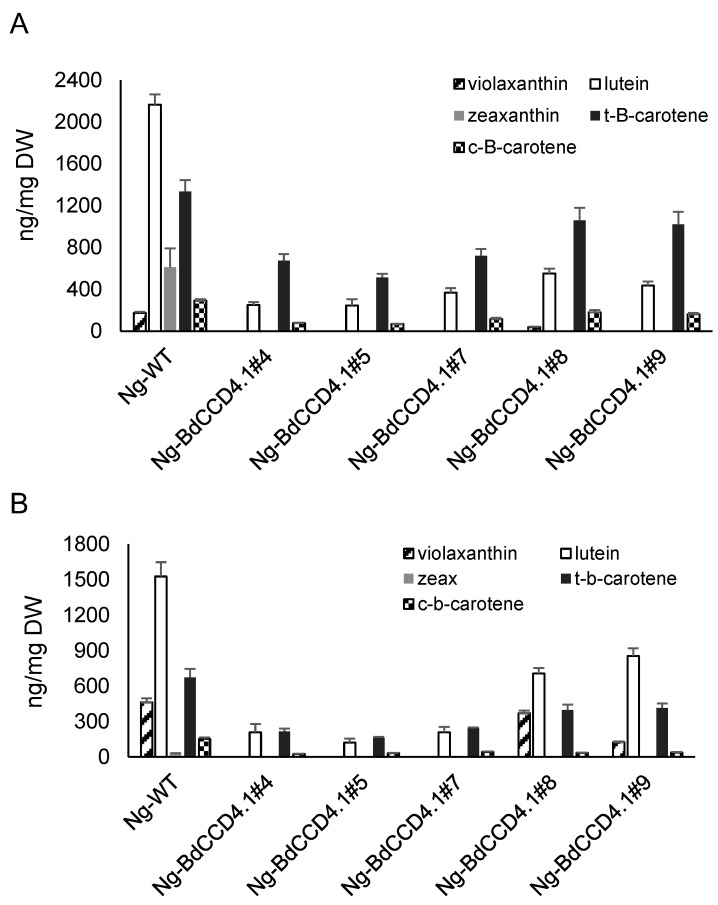
Accumulation of carotenoids in Wt and transgenic T1 lines of *Nicotiana glauca* transformed with BdCCD4.1. (**A**) Carotenoid levels in non-polar extracts of leaves from the Wt and transgenic lines of *N. glauca*. (**B**) Carotenoid levels in non-polar extracts of petals from the Wt and transgenic *N. glauca*. Analyses were conducted in triplicate. Error bars represent the SD. DW, dry weight.

The levels of endogenous carotenoids were analyzed in the leaves and petals of transgenic *BdCCD4.1* plants (Figure 4). Five major carotenoids were detected in the extracts of the control petals and leaves: violaxanthin, lutein, zeaxanthin, all-*trans*-β-carotene, and its geometric isomer 9-*cis*-β-carotene. In all of the transgenic lines, there was a reduction in the content of these carotenoids in the leaves as well as in the petals (Figure 4A,B). This suggests that the production of crocins occurred at the expense of the native carotenoids present in these tissues. In the leaves, zeaxanthin was not detected in the transgenic plants, and the lutein concentration was reduced by 74–92%. For β-carotene, the fluctuations were greater between the different lines, with a 23–70% reduction in its concentration with respect to the WT plants. Similar fluctuations have been previously observed in *N. glauca* plants engineered to produce ketocarotenoids [31]. Interestingly, the lower reduction in the β-carotene levels was observed in those lines accumulating higher levels of crocins (lines #7 and #9, with a 23.6% and 27.4% reduction, respectively). In the petals, a reduction in concentration was also observed for lutein and β-carotene, whose levels fluctuated within broad ranges of 44–92% and 45–71% for lutein and β-carotene, respectively. As observed in the leaves, a lower reduction was observed for lines #7 and #9, which showed higher levels of crocins in the petals (Figure 2).

### 2.4. Stability of Endogenous Apocarotenoids in Leaves and Petals of Transgenic N. glauca

**Figure 5:**
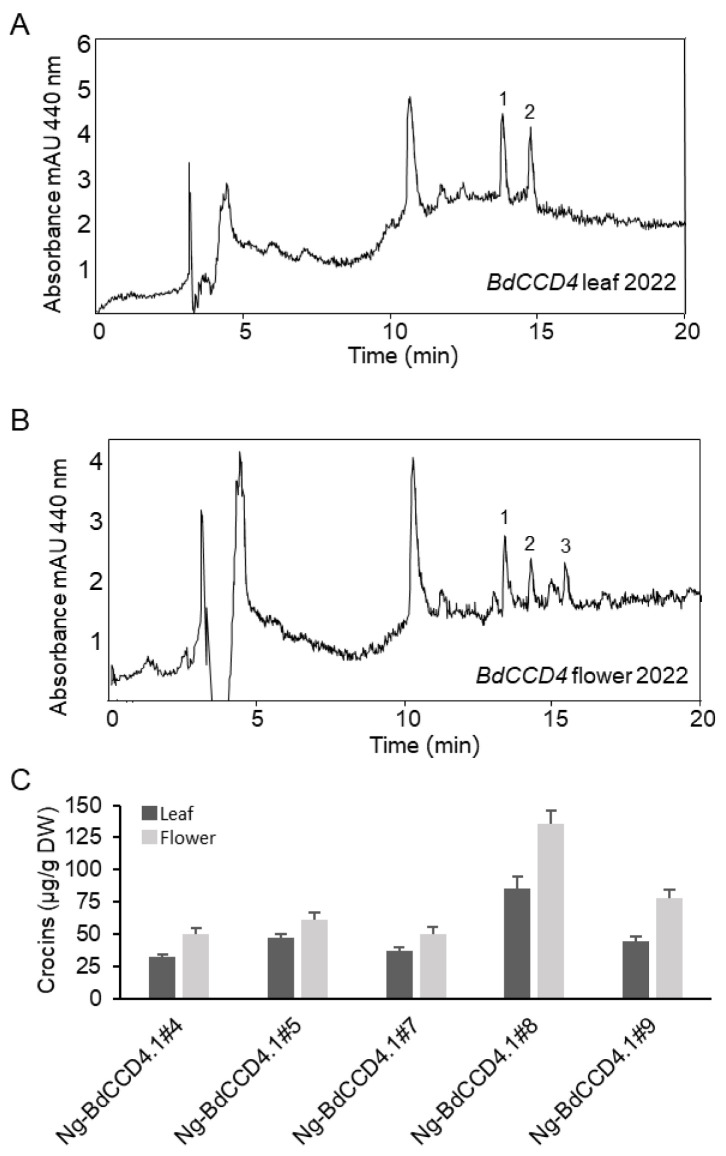
Accumulation of crocins in the Wt and transgenic T1 lines of *Nicotiana glauca* constitutively expressing BdCCD4.1. (**A**) HPLC-DAD analysis of polar extracts from Wt and *N. glauca* leaves at 440 nm. (**B**) HPLC-DAD analysis of polar extracts from transgenic and Wt *N. glauca* petals at 440 nm. Peaks for the abundant crocins in the transgenic lines are denoted by numbers in leaves (1 = trans-crocin 2; 2 = trans-crocin 1) and in petals (1 = trans-crocin 2; 2 = trans-crocin 1; 3 = cis-crocin 2). Crocins were completely absent from the Wt plants. mAU, milli-absorbance units. (**C**) Apocarotenoid accumulation in leaves and petals of transgenic lines. Analyses were done in triplicate. Error bars represent the SD. DW, dry weight.

No information is available on the stability of apocarotenoids during the storage of tissues from transgenic plants. Similar to other carotenoids, crocins, being highly unsaturated, are prone to degradation during storage. However, in contrast to the lipophilic carotenoids, the stability of crocins is largely affected by water [32]. We determined the stability of crocins in lyophilized samples stored at room temperature after a 3-year period. The general chromatographic profile at 440 nm of polar extracts from the leaves and petals of the transgenic samples analyzed after 3 years of storage was clearly different (Figure 5). In general, in the petals and leaves, there was a clear reduction in the content of crocins, and crocins with two and one glucose molecules predominated (Figure 5A,B). In addition, crocin degradation in petals was less pronounced than that in leaves, at 54–60% in petals and 71–83% in leaves.

## 3. Discussion

The production of high-value saffron apocarotenoids in heterologous systems has been reported in bacteria, yeast, and plants with different degrees of success, including tomato, *N. benthamiana*, and *N. glauca* [17,22,23]. Among the different heterologous systems, plants are an attractive production platform due to the presence of the native enzymes for the proper production of carotenoid substrates for the biosynthesis of crocins. Previously, the *BdCCD4.1* gene was transiently expressed in *N. benthamiana* plants using a viral vector [23], resulting in the accumulation of up to 79 ± 8.6 µg/g DW of crocins in leaves. In this study, we aimed to investigate whether the stable expression of *BdCCD4.1* resulted in an increased level of crocins when expressed in *N. glauca* plants. Recently, *N. glauca* and *N. benthamiana* transgenic lines expressing *CsCCD2L* were evaluated for their capacity to produce crocins [22], with transgenic *N. glauca* plants proving more effective, suggesting that, in *N. benthamiana*, other factors may limit crocin accumulation.

We evaluated the levels of crocins in the leaves and petals of control and transgenic lines of *N. glauca* plants. Crocins were only detected in transgenic lines, and in three out of the five lines, higher accumulation of crocins was observed in the leaf tissue, while two lines showed similar levels of crocins in the petals and leaves. However, the accumulated crocins were qualitatively different in terms of their glucosylation patterns. The addition of sugar moieties to the crocetin molecule is mediated in *N. glauca* by unspecific UDP-glycosyltransferases, which increase the solubility of crocins, allowing their transport to the vacuole [33]. Similarly, in *N. glauca* plants modified to produce ketocarotenoids, the levels of ketocarotenoids were higher in the petals than in the photosynthetic tissues, while the profile of the different ketocarotenoids was also different [31]. Interestingly, in transgenic lines expressing *CsCCD2L*, the levels of crocins in the petals were lower than those in the leaves. The higher accumulation in leaves might have been due to the presence of higher levels of the substrate zeaxanthin [26]. Nevertheless, this possible tissue-specific activity of endogenous CCDs in leaves and petals, acting to reduce the carotenoid pool, should also be considered, as well as the availability of free, non-esterified zeaxanthin for crocin biosynthesis.

The stability of bioactive compounds with putative health benefits under different storage conditions is crucial to guarantee their properties for a long period of time, and this is especially relevant for high-value metabolites, such as crocins [34]. Similar to other carotenoids, crocins have a highly unsaturated chemical structure and are consequently susceptible to degradation during storage [35]. They are especially sensitive to heat and light, and their stability is also affected by water [36]. Earlier studies with dry saffron stigma stored at room temperature showed a 20% loss of crocins in a 12-week period (Shahidi et al., 2008), and for standardized extracts kept at 4 °C for 22 months, a 30% loss was reported (Suchareau et al., 2021). Therefore, it is necessary to test the stability of crocins in crocin-rich *N. glauca* plants in order to facilitate future application. Freeze-drying is considered as the most effective method for preserving and maintaining bioactive chemical compounds in plant tissues [37], and is especially recommended for heat-sensitive materials and biotechnological products. Freeze-dried commercial saffron contains higher amounts of safranal and crocin compared with different traditional methods for saffron preservation [38,39]. In this study, crocins were extracted from freeze-dried leaves and petals, and the tissues were stored at room temperature in the dark for a long period of time. After three years of storage, the levels of crocins were reduced by an average of 58.06 ± 2.20% in the petals and 78.37 ± 5.08% in the leaves. The higher stability of crocins in the petals might have been due to the different crocin moieties that accumulated in each tissue. The petals contained crocins with a higher glucosylation pattern, with crocins with three and four glucose molecules, while crocins with two glucose molecules were predominant in the leaves. Although studies on saffron stability showed similar kinetics of degradation for crocins with different degrees of glucosylation, the presence of a greater number of glucose molecules suggested a slower rate of degradation of crocins in the samples [40,41]. In the petals, the crocins with two glucose molecules were in a cis-configuration. Studies on carotenoid isomers have shown that some cis-isomers are more stable than trans-isomers [42], and this could be the case for crocins as well. Previous studies on the stability of β-carotene from Golden Rice during storage indicated losses after six months ranging from 68% to 80%, depending on the processing, temperature, and packing conditions [43]. Similarly, 50 to 65% carotenoid losses have been reported for biofortified maize after 4 to 6 months under conventional storage conditions [44,45].

In conclusion, the results of this study demonstrated that the stable expression of *BdCCD4.1* in *N. glauca* plants allowed the accumulation of up to 321.6 ± 21.3 µg/g DW and 302.7 ± 25.6 µg/g DW of crocins in petals and leaves, respectively, but it was lower in comparison with the expression of CsCCD2L. However, the obtained levels were higher than those previously reported in transient expression experiments in tobacco plants (79 µg/g) [23], or in *N. tabacum* by chloroplast transformation expressing a CCD4 from Bixa Orellana (>50 µg/g) [46], demonstrating that stable expression in *N. glauca* not only affords high levels of crocins, but importantly provides clues about the stability of these compounds in different tissues.

## 4. Materials and Methods

### 4.1. Plant Material

Wild-type (Wt) *N. glauca* and transgenic plants were grown under controlled growth conditions with a 25/20 °C day/night temperature cycle, a 12 h photoperiod (mean irradiance of 100 μmol m^−2^ s^−1^), and 60–90% relative humidity. Mature leaves (the 5th and 6th leaves) were collected from 5 WT and 5 transgenic plants for each line, frozen in liquid nitrogen, and stored at −80 °C until use.

### 4.2. Vector Construction

The plasmid for transformation was created with the BdCCD4.1 gene. The CaMV35S promotor was used to control the expression of the transgene. The Goldenbraid strategy was followed to construct the vectors (Sarrion-Perdigones et al., 2014; Sarrion-Perdigones et al., 2013). Briefly, the complete open reading frame (ORF) of BdCCD4.1 was domesticated by removing *Bsm*BI and *Bsa*I in the original sequence using the primers. The product was cloned in the vector pUPD2 of the Goldenbraid modular cloning system. The resulting plasmid pUPD2-BdCCD4.1 was then used to construct the recombinant binary vector: pDGB3Ω1[p35S:BdCCD4.1:T35S-pNos:Hyg:T35S].

### 4.3. Transformation of Agrobacterium and N. glauca

*Agrobacterium tumefaciens* strain LBA4404 was used for transformation by electroporation with the construct pDGB3Ω1[p35S:BdCCD4.1:T35S-pNos:Hyg:T35S]. Transformants were selected on YEB agar plates containing 100 μg/mL rifampicin, 50 μg/mL spectinomycin, and 25 μg/mL gentamicin. *Agrobacterium*-mediated transformation of *N. glauca* was performed according to the leaf disc method [47]. For the selection of transformants, hygromycin B at a concentration of 50 μg/mL was used.

### 4.4. Genomic DNA Isolation and PCR conditions

A PCR reaction with primers specific to *BdCCD4.1* was conducted to verify the presence of the transgene. Genomic DNA was extracted from *N. glauca* leaves using the DNeasy Plant Mini Kit (Qiagen, Hilden, Germany). The amplification conditions were as follows: 95 °C for 2 min, followed by 35 cycles at 95 °C for 20 s, 55 °C for 20 s, and 72 °C for 1 min per kilobase, followed by a final extension at 72 °C for 5 min. The PCR products were separated and visualized on 0.8% agarose gel stained with ethidium bromide using a UV transilluminator.

### 4.5. Carotenoid and Apocarotenoid Extraction and Quantification

Polar and non-polar metabolites were extracted from 50 mg of lyophilized petals and leaves. For the analysis of crocins, leaves were extracted in cold 50% methanol with shaking. The soluble fractions were analyzed by HPLC-DAD as previously described [23]. Carotenoids were extracted with 1:2 cold extraction solvents (50:50 methanol:CHCl_3_) and analyzed using an Agilent 1100 Series HPLC system with a YMC ODS-A 250 × 4.6 mm column. The separation, identification, and quantification of pigments were carried out as described previously [23]. Peaks were detected by their absorption at 455 nm, and individual compounds were identified and quantified by comparison with known amounts of pure standards, as previously reported [48]. All samples were analyzed in triplicate.

## Data Availability

The data presented in this study are available in article.

## Abbreviations

Geranylgeranyl diphosphate (GGDP), Carotenoid cleavage dioxygenase (CCD). Abscisic acid (ABA), Hygromycin B phosphotransferase (Hph), Wild type (Wt).
